# Acetylation and deacetylation in cancer stem-like cells

**DOI:** 10.18632/oncotarget.19167

**Published:** 2017-07-11

**Authors:** Na Liu, Shiqi Li, Nan Wu, Kin-Sang Cho

**Affiliations:** ^1^ Department of Ophthalmology, Southwest Eye Hospital, Southwest Hospital, Third Military Medical University, Chongqing, China; ^2^ Center of biotherapy, Southwest Hospital, Third Military Medical University, Chongqing, China; ^3^ Schepens Eye Research Institute, Massachusetts Eye and Ear Infirmary, Harvard Medical School, Boston, Massachusetts, USA

**Keywords:** cancer stem cell, acetylation, deacetylation, HDAC, HAT

## Abstract

Cancer stem-like cell (CSC) model has been established to investigate the underlying mechanisms of tumor initiation and progression. The imbalance between acetylation and deacetylation of histone or non-histone proteins, one of the important epigenetic modification processes, is closely associated with a wide variety of diseases including cancer. Acetylation and deacetylation are involved in various stemness-related signal pathways and drive the regulation of self-renewal and differentiation in normal developmental processes. Therefore, it is critical to explore their role in the maintenance of cancer stem-like cell traits. Here, we will review the extensive dysregulations of acetylation found in cancers and summarize their functional roles in sustaining CSC-like properties. Additionally, the use of deacetyltransferase inhibitors as an effective therapeutic strategy against CSCs is also discussed.

## INTRODUCTION

Since being proposed in 1983 [1], the theory of cancer stem-like cells (CSCs) has been gradually constructed and publicized. According to the CSC hypothesis, tumors are organized hierarchically; the cancer cell population at the top of the hierarchy displays stem cell properties and sustains tumorigenesis. A growing body of evidence supports the pivotal role of CSCs in pathological self-renewal, drug-resistance and cellular heterogeneity of cancer. Bonnet and Dick first isolated CD34^+^CD38^-^ cells that are capable of initiating human acute myeloid leukemia (AML). Human AML is organized hierarchically and originates from primitive hematopoietic cells [2]. Subsequent studies have revealed the presence of tumor initiating cells in solid tumors such as breast [3], brain [4], colon [5, 6], prostate [7] and liver [8] cancers. Although several unanswered questions like unknown CSCs universality, variable frequency and non-universal markers [9] continue to persist, the CSC model is a tempting approach to investigate tumor initiation and progression.

The CSCs may either arise from dysregulated normal stem cells or from mature cells which have de-differentiated into a stem-like state. Increasing evidence points to the epigenetic aberration in normal developmental processes as a key driver of CSC-like properties [10–12]. Acetylation is one of the most important protein modifications, which occurs via a dynamic process regulated by the balance between histone acetyltransferases (HATs) and deacetylases (HDACs). Acetylation and deacetylation influence the plasticity of chromatin structure by changing the electrical property of acetylated sites of histone and improve the stability of many non-histone proteins by covering ubiquitination sites [13]. Through these regulatory mechanisms, acetylation and deacetylation participate in the modulation of expression of various genes, which in turn modulates cellular activities like proliferation, differentiation and migration. Clarifying the result of acetylation disorders and investigating how these changes modify normal cellular activities and maintain the CSCs-related “stemness” may provide insights into epigenetic modification of CSCs and tumorigenesis.

In this review, we summarize the recent advances in the understanding of the roles of acetylation and deacetylation in CSCs, laying stress on the effect of the family of HATs and HDACs on CSCs and the potential clinical application of HDAC inhibitors to eliminate CSCs.

### Classification of HATs and HDACs

Histone acetyltransferases (HATs) refer to a group of enzymes which are responsible for both histone and non-histone acetylation. Acetylation on histone tail is important for histone assembly into nucleosomes by histone chaperones, and determines whether it can create an open and permissive chromatin environment for transcription. Briefly, HAT activity/acetylation relaxes the chromatin structure, facilitates transcriptional activity and hence, increases gene expression. Extensive research has been performed on the acetylation of lysine residues on histone 3 (H3) and 4 (H4). Acetylation of Lysine 56 (K56) in the helical core of H3 opens yeast chromatin and enables gene transcription, DNA replication and repair, and prevents epigenetic silencing [14]. Acetylation of H4 at K16 regulates chromatin compaction and folding [15]. Besides histones, p53 was the first non-histone protein found to be acetylated by HATs [16]. A growing number of non-histone proteins have been identified as acetylation targets. Reversible acetylation of these proteins has been shown to play a key role in DNA binding affinity, transcriptional activation, protein stability and protein-protein interactions [17]. Although acetylated proteins were shown to increase DNA binding affinity and transcriptional activation, acetylated YY1 and ER alpha proteins are notable exceptions in this respect [13].

HATs are categorized into 2 types based on their cellular location (Table 1). Type A HATs contain a number of heterogenic enzymes in the nucleus. These exhibit global functional similarity with respect to catalysis of transcriptional processes. Type B HATs are cytoplasmic proteins responsible for acetylation of newly synthesized histone proteins. Based on their structural homology and the mechanism of acetyl transfer, Type A HATs members are classified into five distinct families with different targets and functions: GNAT family, p300/CBP family, MYST family, basal TF family, and NRCF family [18]. Family of p300/CBP has more than 75 protein substrates [19]; p300/CBP-mediated acetylation is important for p53 function that is believed to be related to carcinogenesis [20]. Members of MYST family are responsible for DNA repair and gene silencing [21]. In addition, Type B HATs are further divided into HAT1, HAT2, HatB3.1, Rtt109, and HAT4 [22].

**Table 1:** Classification of histone acetyltransferases and deacetylases

	Members	Location
**HATs**		
** Type A**	GNAT, p300/CBP, MYST, basal TF, and NRCF	Nucleus
** Type B**	HAT1, HAT2, HatB3.1, Rtt109, and HAT4	Cytoplasm
**HDACs**		
** Class I**	HDAC1, HDAC2, HDAC3, HDAC8	Nucleus
** Class II**	II a: HDAC4, HDAC5, HDAC7, HDAC9	Nucleus/cytoplasm
	II b: HDAC6, HDAC10	Mainly cytoplasm
** Cass III**	SIRT1-7	Nucleus/cytoplasm
** Class IV**	HDAC11	Nucleus/cytoplasm

Histone deacetyltransferases (HDACs) are a family of enzymes which function antagonistically to HATs. Acetylation levels of histone and non-histone proteins are determined by the activities of HATs and HDACs. In contrast to the role of HATs, HDACs are thought to act as transcriptional corepressors [23, 24]. HDACs are believed to be bound to repressed genes and are replaced by HATs once these genes need to be activated [25]. Deacetylation on lysine residues of histone, induced by HDACs, is associated with a condensed structure of chromatin and limited accessibility of the transcription machinery, and hence leads to gene silencing [26, 27]. Besides modifying acetylation on histone protein, HDACs also regulate gene expression by deacetylation of non-histone proteins. For example, HDACs were shown to directly act on a series of transcription factors such as p53, HMG proteins, STAT3, c-MYC, E2F, and NF-kB [13, 28]. Deacetylation by HDACs contributes to degradation of many proteins, which is a prerequisite to subsequent ubiquitination.

According to their homology with yeast HDACs, mammalian HDACs are classified into four classes (class I to IV). Class I HDACs are localized in nucleus and are homologous to Rpd3 in yeast; these include HDACs 1, 2, 3, and 8. Class II HDACs are homologous with yeast Hda1 and are further subdivided into IIa (HDAC 4, 7, and 9) and IIb (HDAC 6 and 10). Class II HDACs are able to shuttle between nucleus and cytoplasm. Thus, class II HDACs may deacetylate non-histone proteins in cytoplasm. Class III HDACs, also referred to as sirtuins (SIRT1-7), are homologous to the yeast Sir2 family proteins. Class III HDACs require coenzyme NAD^+^ for activation [29]. In contrast to the other HDAC classes, Class IV HDACs have only one member (HDAC 11) that is homologous to both class I and class II HDACs.

### Dysregulation of HATs in cancers

Increasing evidence points to dysregulation of HATs as a driving force of cancer. HAT activities are interfered as a consequence of various genetic or epigenetic alterations in several malignancies. HATs have been suggested to play a dual role in carcinogenesis; these could either function as tumor suppressors (by inhibition of cell proliferation) or act as oncogenes (by activation of malignant proteins via abnormal acetylation [30]. Long *et al.* suggested that cancer is associated with globally hypoacetylated chromatin. Increased histone acetylation induced by short chain triglyceride glyceryl triacetate (GTA) was shown to arrest growth of oligodendroglioma derived cells in the G0 phase without affecting normal cells [31]. Interestingly, recent studies confirmed that alcohol exposure can alter histone acetylation pattern and that this might contribute to liver cancer [32].

Roles of HATs in tumorigenesis might depend upon the site of acetylation of proteins as well as on the type of cancer. Deficient in acetylation of H3 was detected in patients with prostate cancer [33]. Histone hyperacetylation of H3K56 has also been observed in hepatocellular carcinoma [34]. Kang *et al.* suggested that HTAs could be a potential therapeutic target for cancers. They demonstrated that curcumin-induced hypo-acetylation leads to apoptotic cell death in brain cancer [35]. Roche *et al.* reported higher H3K27 acetylation level in the tumor compartment as compared to that in the corresponding stroma, in their study on lung cancer [36]. Inhibition of LDH-A enzyme activity and the consequent decrease in LDH-A protein level was shown to reduce K5 acetylation of lactate dehydrogenase A (LDH-A) in a study on human pancreatic cancers [37]. Upregulated expression of global transcriptional co-activator p300 in prostate cancer tissues has also been reported [38]. Inactivation of the p300 gene has been implicated in the development of colorectal, gastric, breast and brain cancers [39, 40].

### Roles of HATs in normal stem cells and CSCs

HATs and their cofactors are known to be involved in the modulation of self-renewal and differentiation ability of stem cells [41]. Mof (a member of MYST family) was reported to play an essential role in the maintenance of ESC self-renewal and pluripotency. ESCs with Mof deletion were shown to have aberrant expression of stemness-related core transcription factors including Nanog, Oct4, and Sox2 [42]. Akt was shown to modulate the stemness of induced pluripotent stem cells (iPSCs) by facilitating the p300-mediated acetylation of Oct4, Sox2 and Klf [43]. MYST family has been shown to play a central role in stem cell function and development [21]. In an experimental study by Liu *et al.*, sodium butyrate (NaB) promoted the differentiation of bone marrow-derived mesenchymal stem cells (MSCs) into smooth muscle cells (SMCs) in rat through histone acetylation. Further, NaB induced upregulation of H3K9 and H4 acetylation and enhanced expression of target genes [44]. Acetylation of H3K56 is linked to the core transcriptional network in human embryonic stem cells [45]. Interestingly, acetylation of lysine residue of Sox2 was shown to be associated with its nuclear export in embryonic stem cells. Blockade of acetylation led to Sox2 retention in the nucleus and sustained the expression of its target genes under hyperacetylation or differentiation conditions [46]. This finding suggests a regulatory mechanism for acetylation-related cell stemness.

Till now, the relationship between HATs and CSCs has mainly been studied in the context of hematological malignancies [47, 48]. CBP/p300 is a key co-activator for the transforming capacity of transcription factor c-Myb. Several studies have shown that interaction of p300/CBP with c-Myb is required for self-renewal and malignant transformation of leukemia stem cells malignant transformation [49–51]. AML1-ETO fusion protein, a transcription factor which is crucial for leukemogenesis, is acetylated by p300 in leukemia cells. Inhibition of p300 downregulated acetylation of AML1-ETO and impaired the self-renewal ability of leukemia stem cells [52, 53]. Human monocytic leukemia zinc-finger protein MOZ and its paralog MORF belong to the family of MYST. The MOZ fusion protein MOZ-TIF2 was shown to interact with transcription factor PU.1 to activate the expression of CSF1R. In a mouse model, Aikawa *et al.* showed that PU.1-mediated upregulation of CSF1R is crucial for the establishment and maintenance of leukemia stem cell induced by MOZ-TIF2; this indicates the potential use of CSF1R inhibitors as an effective leukemia stem cell targeting therapeutic approach [54]. Moreover, MOZ-TIF2 can also cooperate with FLT3–ITD mutation to transform hematopoietic cells which results in an increase in the number of leukemic stem cells. STAT5 signaling is necessary to maintain the self-renewal property of leukemia stem cells in MOZ–TIF2 driven leukemia [55].

### Dysregulation of HDACs in cancers

The relation between abnormal activities of HDACs and carcinogenesis has drawn a lot of attention. Aberrant recruitment of HDACs to specific promoters has been shown to be associated with carcinogenesis [56, 57]. Similar to HATs, HDACs also function as a double-edged sword and is thought to be dosage-determinant in tumorigenesis [58–60]. Histone hypoacetylation or loss of acetylation at H4K16 and trimethylation at H4K20 are commonly observed in cancer patients. In teratoma, loss of HDAC1 was shown to increase both apoptosis and cell proliferation [61]. A significant upregulation of histone deacetylases activity has been determined in prostate cancer cells [33]. In human skin cancer cells, SIRT2 is downregulated and deficiency of SIRT2 was shown to promote tumor growth in mice [62]. Yasui *et al.* reported reduced expression of H4 acetylation in gastric cancer patients, which suggested that reduced histone acetylation is associated with the depth of tumor invasion and nodal metastasis of gastrointestinal cancers [63]. Moreover, overexpression of HDAC2 has also been found in colorectal cancer [64].

### Role of HDACs in normal stem cells and CSCs

HDACs also participate in the regulation of stemness property of normal stem cells. HDAC1 and HDAC3 were shown to be activated by hypoxia and mediate the differentiation of mouse embryonic stem cells and hematopoietic stem cells [65]. Octamer-binding transcription factor 4 (Oct4) is an important transcriptional factor of embryonic stem cells which is commonly used as a marker of normal stem cells and cancer stem-like cells. HDAC1 was shown to repress the expression of Oct4 in cervical cancer cells [66]. HDAC1 and HDAC2 are essential for maintaining the homeostasis of hematopoietic cells. Inhibition of both HDAC1 and HDAC2 lead to the loss of hematopoietic stem cells [67]. In addition, a recent study demonstrated that HDAC1 and HDAC2 play a critical role in regulating self-renewal ability of stem cells by maintaining the expression levels of key pluripotent transcription factors. Reduced expression of pluripotent transcription factors such as Oct4, Nanog, Esrrb, and Rex1 was detected after suppression of HDAC1 and HDAC2 activities [68]. However, the functional role of SIRT1 in stem cells is not clear. Ma *et al.* reported that SIRT1 suppressed self-renewal of adult hippocampal neural stem cells. They found an increase in SIRT1 expression during adult hippocampal neural stem cells differentiation as well as a promotion of proliferation and self-renewal rates in adult hippocampal neural stem cells of SIRT1 knockout (KO) mice [69]. However, in bone marrow-derived mesenchymal stem cells, SIRT1 was reported to maintain self-renewal and multipotency by directly regulating SOX2. RNA interference of SIRT1 downregulated SOX2 expression, which impaired the self-renewal and differentiation capacities of human bone marrow (BM)-derived MSCs [70]. SIRT1 could deacetylate retinoic acid binding protein II (CRABPII). Deficiency of SIRT1 was shown to cause accumulation of CRABPII in nucleus, which enhanced homeostatic retinoic acid (RA) signaling and accelerated embryonic stem cell (mESC) differentiation in response to RA [71]. Besides, deacetylation of β-catenin by SIRT1 was shown to inhibit its nuclear accumulation and induce transcription of genes for MSC differentiation [72]. Oct4 was reported to inactivate p53 through SIRT1-mediated deacetylation, which maintained the pluripotency of human embryonic stem cells [73]. Under conditions of cytokine-induced proliferative stress, ablation of SIRT1 was shown to promote proliferative expansion of hematopoietic progenitor cells; the underlying molecular mechanism is possibly linked to increased Hoxa9 expression [74].

Many studies have revealed the role of HDACs in CSCs. HDAC3 was found to be highly expressed in liver CSCs and its expression was significantly correlated with both Nanog and CD133. Knock-down of HDAC3 using specific inhibitors suppressed both sphere and clone formation efficiency accompanied with decreased expression of stem cell markers like Nanog, Oct4 and SOX2 [75]. During oncogenic transformation of neural stem cells, SIRT1 is required for the survival of glioma stem cells via a p53-dependent manner [76]. In colorectal CSC-like cells, high expression level of SIRT1 was detected with co-localization of CD133 and SIRT1. SIRT1 deficiency was accompanied with decreased percentage of CD133^+^ cells, attenuated ability for colony and sphere formation and inhibited tumorigenicity. Furthermore, expression of other stemness-associated genes including Cripto, Nanog, Oct4, Tert and Lin28 were reduced by SIRT1 knockdown [77]. Previous studies showed that SIRT1 repressed the activity of p53 by deacetylating the C-terminal Lys120, Lys164 and Lys382 residues [78–80]. In chronic myelogenous leukemia (CML), pharmacological or genetic inhibition of SIRT1 was shown to increase apoptosis in leukemia stem cells [81], which indicates that activation of p53 via SIRT1 inhibition is a feasible approach to target CML stem cells. K382 of p53 could be deacetylated by SIRT1. SIRT1 inhibition could increase the transcriptional activity of p53 which results in the increased expression of several p53 target genes including Bax, Necdin and Gfi-1 in CML CD34^+^ cells. Choudhary *et al.* showed that other than p53, several key p53-related proteins such as DAXX, PML, PTEN, and HAUSP are also acetylated [82]. It is suggested that these members in the p53 circuitry may also contribute to the effects of SIRT1 inhibition. However, SIRT1 knockdown did not increase the expression of p21 in CML progenitors, which suggests that some other pathways may counteract the effects of p53 acetylation on p21 induction [83]. Similarly, Zeisig *et al.* demonstrated the same role for SIRT1 inhibition in eradicating FLT3-ITD AML stem cells, possibly through a positive feedback loop with c-MYC [84]. Moreover, a very recent study confirmed the increase of HDAC in cisplatin-enriching CSCs of non-small cell lung cancer. The authors further reported that combination of HDAC inhibitor and cisplatin reinforced the antitumor effect, both *in vitro* and *in vivo* [85].

### HDAC Inhibitors as anti-cancer agents

Histone deacetylase inhibitors (HDACIs) are currently being examined in clinical trials against cancer and other diseases like rheumatoid arthritis [86]. These small molecules are able to mediate the induction of both apoptosis and autophagy, which may be a mechanism of anticancer activity in a variety of cancer cell lines [87]. Although the specific anticancer molecular mechanism still remains to be investigated, emerging evidence suggests that inhibition of HDACs may have growth-inhibitory and differentiation driven effects on tumors [88]. HDACIs are currently well recognized as anticancer agents. FDA has approved two HDACIs (vorinostat and romidepsin) for clinical use, while many others are currently being clinically assessed [89]. HDACIs can be classified into several groups based on their chemical structure: cyclic peptides (apicidin, romidepsin); hybrid molecules; hydroxamic acids (trichostatin A, vorinostat); carboxylic acids (valproate, butyrate); aminobenzamides (entinostat, mocetinostat); and epoxyketones (trapoxins). According to the specificity of these HDACIs, they could also be divided into three groups: nonselective HDACI; selective HDACI; and multi-pharmacological HDACI [90]. To date, many of HDACIs have undergone clinical development, either alone or in combination with other anticancer agents [91]. It is worth noting that efficiency of pharmacological inhibition with HDACs was shown to be comparable to that of genetic knockdown/knockout, especially in terms of its effects on multi protein complex formation and function [92].

### Anti-CSCs potential of HDACI in cancer treatment

Many studies have revealed the role of HDACIs in the regulation of self-renewal and differentiation. Trichostatin A (TSA) treated bone marrow-derived multipotent adult progenitor cells (MAPC) showed at least a three-fold increase in the expression of endothelial cell (EC) marker genes VE-cadherin, Flk1 and vWF. It is suggested that HDACI enhanced the differentiation of MAPC to EC [93]. Class I HDACI vorinostat (SAHA) and class II HDACI sodium butyrate were both shown to suppress the formation of neurospheres in adult mouse neural stem cells by arresting cell cycle in the G1 phase [94]. Furthermore, Legartova *et al.* observed enterocytic differentiation of colon cancer cells induced by sodium butyrate with increased mono-, di-, and tri-acetylation of histone H2B and a significant upregulation in di- and tri-acetylation of histone H4 [95]. These findings suggest the potential role of HDACIs in the induction of stem cell differentiation. However, another HDACI valproic acid (VPA) was previously reported to enhance self-renewal and cytokine-induced expansion of hematopoietic stem cells [96–98]. VPA could maintain the self-renewal ability of mouse embryonic stem cells (mESCs) under hypoxic conditions by suppression of HIF-1alpha [65]. Conversely, in a recent study, VPA induced significant upregulation of neuroprogenitor marker Musashi, CD133 and Nestin, which suggests that VPA may play a key role in neuronal differentiation of human bone-marrow mesenchymal stromal cells (BM-MSCs) [99].

**Figure 1:**
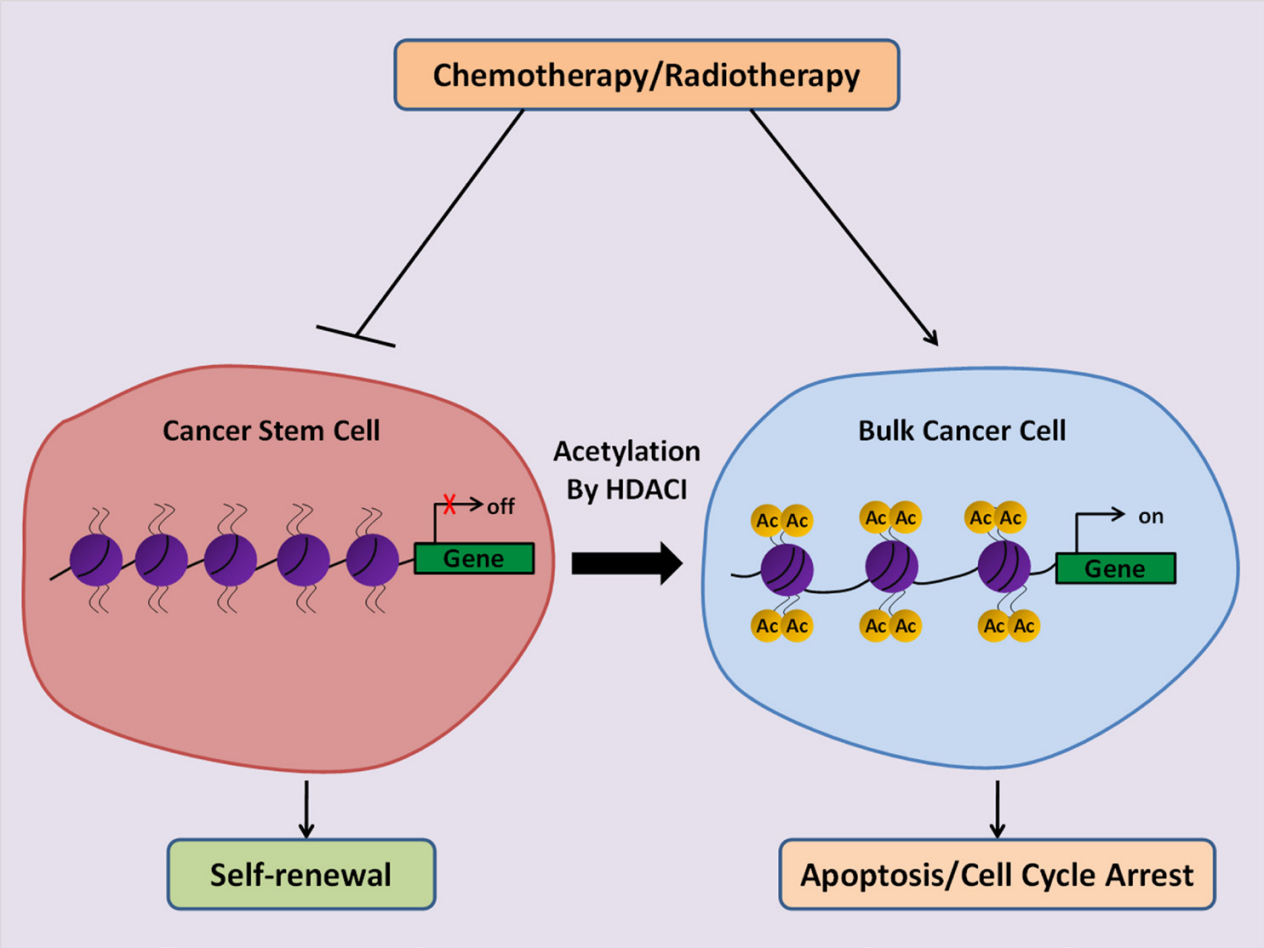
Schematic illustration of the anti-cancer potential of HDACIs to eliminate CSCs CSCs are resistant to conventional therapies such as chemotherapy and radiation which makes the cancer incurable. Therapeutic strategy using HDACIs induces differentiation of CSCs towards cancer cells by activation of gene transcription, and thus renders the tumors sensitive to therapies.

Compelling evidence suggests that CSCs are responsible for chemotherapy and radiotherapy resistance of cancer. It is, therefore, a great concern to confirm if HDACIs could specifically target CSCs to increase the therapeutic efficacy of conventional treatment (Figure 1). Most studies have shown promising results of HDACIs in suppressing CSCs expansion and tumor aggressiveness [100]. Treatment of pancreatic CSCs with SAHA resulted in reduced self-renewal capacity and induction of apoptosis through inhibition of Notch pathway [101]. Vorinostat was shown to increase the sensitivity of SK-N-Be(2)C-resistant human neuroblastoma cells to chemotherapy and led to the loss of tumor sphere forming ability, reduced invasion and the side population percentage. Nine stemness-linked genes including *ERCC5, ABCB1, ABCC4 S100A10, LMO2, SOX2, IGFBP3, TCF3* and *VIM* were found to be downregulated in the presence of vorinostat [102]. Sodium butyrate (NaB) was shown to promote neuronal differentiation of medulloblastoma (MB) cells through upregulation of neuronal differentiation marker Gria2 at the transcriptional level [103]. Similar to MB, treatment of endomertrial cancer cells with NaB induced production of intracellular reactive oxygen species (ROS) and DNA damage which had an inhibitory effect on the proliferation of endometrial CSCs [104]. Frame *et al.* showed that the HDACI Trichostatin A sensitized prostate CSCs to radiation [105]. Pathania *et al.* confirmed that a combination of the DNMT inhibitor, 5-azacytidine, and the HDAC inhibitor, butyrate A, markedly suppressed the tumorigenicity of CSCs and attenuated the growth of breast tumor [106]. In addition, a very recent study demonstrated synergistic cytotoxic effects of combined HDACI HNHA and sorafenib therapy against CSCs of anaplastic thyroid cancer [107]. These findings suggest that HDACIs may considerably increase the efficiency of conventional therapies by driving the differentiation of CSCs. However, some papers revealed inconsistent findings that HDACI may also stimulate the dedifferentiation of cancer cells through activation of developmental signaling pathways [108–110]. This discrepancy may reflect the complexity of epigenetic regulation in cancers. The precise mechanism of HDACI in CSC regulation deserves to be further investigated.

## PERSPECTIVE

Based on the available evidence, it is difficult to conclude that acetylation and deacetylation merely serve to enhance or inhibit CSCs. However, a vast majority of studies do support the anti-cancer role of acetylation and the sustainer role of deacetylation in cancer stemness regulation. Numerous studies have confirmed HDACIs as powerful therapeutic agents against cancer which act by promoting differentiation of CSCs. Considering the differences in acetylation levels and sites of acetylation or deacetylation, the efficacy of HDACI may vary in different cancer types or even vary from one patient to another.

The specific molecular mechanism by which acetylation and deacetylation modulate the self-renewal, proliferation, multipotency, metastasiss and drug-resistance of CSCs still remain largely unknown. Combination of different site-specific acetylation and deacetylation may explain the diverse or even paradoxical regulation of HATs and HDACs in CSCs. Thus, the role of individual and combined site(s) specific acetylation or deacetylation is worthy of investigation.

Another largely unexplored field is the relationship between histone and non-histone acetylation. As discussed above, histone acetylation by HATs is associated with transcriptional activation while histone deacetylation by HDACs is involved in transcriptional inhibition. Nevertheless, the functions of non-histone acetylation and deacetylation tend to vary and are mostly uncertain. The role of acetylation/deacetylation on histone and non-histone proteins constitutes the final role of this post-translational protein modification. Thus, further dissecting the specific combinations of sites of acetylation/deacetylation on histone and non-histone proteins may delineate the mechanisms of the paradoxical situation described above.

With regard to HDAC inhibitors, studies suggest that HDACIs act on both CSCs and the rest of bulk cancer cells. HDACIs could drive differentiation of CSCs, inhibit their self-renewal ability, enhance their sensitivity to chemo/radiotherapy or even induce their death. However, before applying HDACI to patients, there are still a lot of hurdles that need to be overcome. The main problem is that the effects of HDACIs are not equivalent to those of HDAC-knockoff. Since HDAC can form HDAC-complexes with other molecules, it is hard to identify the target sites inhibited by HDACIs. Most of the currently used HDACIs are broad-spectrum inhibitors which inhibit many types of HDACs. Therefore the therapeutic results of these HDACIs could not be predicted precisely. The reports that HDACIs may induce dedifferentiation of cancer cells and expansion of CSCs under some circumstances remind us to use HDACIs cautiously in cancer therapy and for treatment of other diseases. Novel HDACIs which target specific HDACs need to be developed and investigated.
